# Supplementary figures and images for: Particle-Based Imaging Tools Revealing Water Flows in Maize Nodal Vascular Plexus
Source: Plants (Basel). 2022 Jun 8;11(12):1533. doi: 10.3390/plants11121533 (PMC9228485; doi:10.3390/plants11121533)

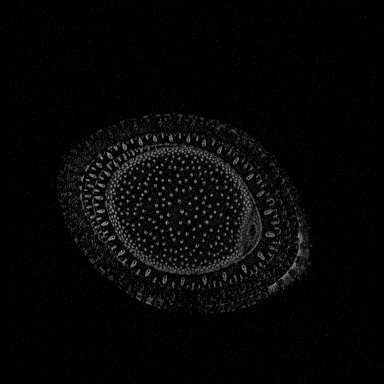

Supplement: Supplementary file 1 [file plants-11-01533-s001.zip › Figure S1.tif]

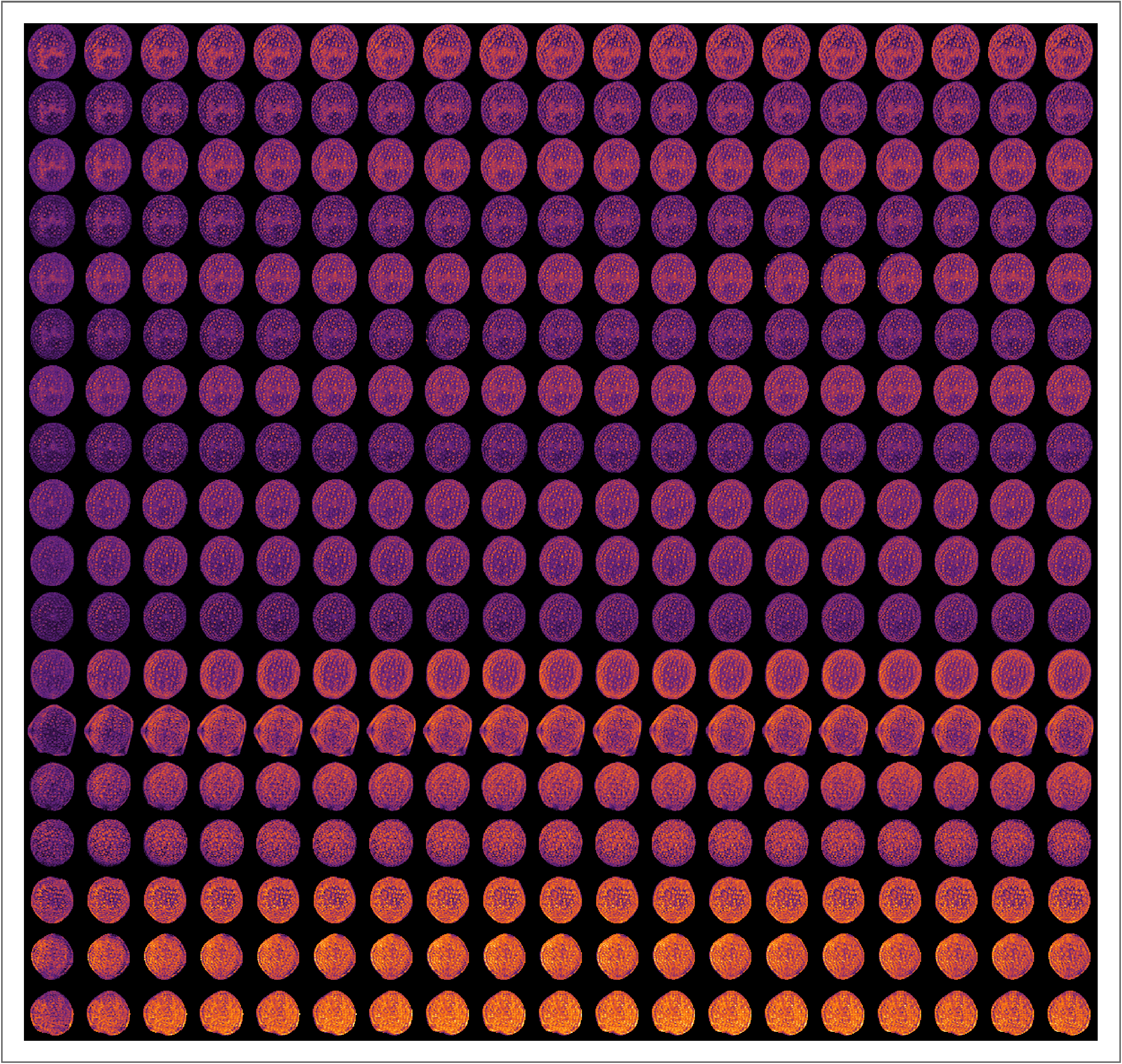

Supplement: Supplementary file 1 [file plants-11-01533-s001.zip › Figure S2.tif]
